# Evaluation of Hepatoprotective Activity of* Adansonia digitata* Extract on Acetaminophen-Induced Hepatotoxicity in Rats

**DOI:** 10.1155/2016/4579149

**Published:** 2016-03-16

**Authors:** Abeer Hanafy, Hibah M. Aldawsari, Jihan M. Badr, Amany K. Ibrahim, Seham El-Sayed Abdel-Hady

**Affiliations:** ^1^Department of Pharmacology and Toxicology, Faculty of Pharmacy, King Abdulaziz University, Jeddah 21589, Saudi Arabia; ^2^Department of Pharmaceutics, Faculty of Pharmacy, King Abdulaziz University, Jeddah 21589, Saudi Arabia; ^3^Department of Natural Products and Alternative Medicine, Faculty of Pharmacy, King Abdulaziz University, Jeddah 21589, Saudi Arabia; ^4^Department of Pharmacognosy, Faculty of Pharmacy, Suez Canal University, Ismailia 41522, Egypt

## Abstract

The methanol extract of the fruit pulp of* Adansonia digitata *L. (Malvaceae) was examined for its hepatoprotective activity against liver damage induced by acetaminophen in rats. The principle depends on the fact that administration of acetaminophen will be associated with development of oxidative stress. In addition, hepatospecific serum markers will be disturbed. Treatment of the rats with the methanol extract of the fruit pulp of* Adansonia digitata *L. prior to administration of acetaminophen significantly reduced the disturbance in liver function. Liver functions were measured by assessment of total protein, total bilirubin, ALP, ALT, and AST. Oxidative stress parameter and antioxidant markers were also evaluated. Moreover, histopathological evaluation was performed in order to assess liver case regarding inflammatory infiltration or necrosis. Animals were observed for any symptoms of toxicity after administration of extract of the fruit pulp of* Adansonia digitata *L. to ensure safety of the fruit extract.

## 1. Introduction

Modern food styles, excessive medications, and exposure to pollutants besides many other factors have led to many serious diseases including liver damage [1]. Production of reactive oxygen species is considered as a crucial factor leading to oxidative damage of tissues. They react with cell membrane; accordingly, many clinical disorders could be attributed to these free radicals [2]. Recently, herbal products have gained attention as a major part of alternative medicine [3, 4]. It is reported that a significant percentage of population depend on natural derived medicines for maintaining health and treatment of diseases [5]. Nowadays, discovery of new drug leads seems to focus on those of plant origin. Herbal drugs play a significant role in the regeneration of liver cells and acceleration of healing process and hence management of many liver disorders [2]. One of the comprehensive examples is silymarin isolated from* Silybum marianum*. Silymarin is a mixture of phenolic compounds (flavonolignans) well known for their radical scavenging activities and thus plays a role in prevention and treatment of oxidative damage caused by reactive oxygen species. Nowadays, silymarin is considered as a major component of many important pharmaceutical preparations in the market introduced for treatment of liver diseases [2, 6, 7]. Based on the fact that a tremendous number of plants could be considered as a gold mine for discovery of hepatoprotective agents, we launched our study in a trial to investigate the fruit pulp of* Adansonia digitata *L.

Our aim here is to discover naturally derived therapeutic agents with hepatoprotective effect. Our work will focus on* Adansonia digitata *L. (commonly known as baobab) which is a tree native to Central Africa and belongs to family Malvaceae. The pulp of baobab fruit is a very important food. It is used by dissolving in milk or in water. This solution is used as a drink, in baking, or as a sauce for food. The pulp is considered as a popular ingredient in ice products [8]. Previous chemical investigation reported that* A. digitata *accumulates flavonoids, terpenoids, steroids, amino acids, vitamins, lipids, and carbohydrates [9]. The dry fruit pulp possesses high percentage of carbohydrates, thiamine, nicotinic acid, and vitamin C; additionally, it reveals potent free radical scavenging activity [8, 10, 11]. Previous reports also indicated that the fruit pulp extract exhibited numerous activities such as anti-inflammatory, analgesic, and antipyretic activities [12]. Since free radical scavenging and anti-inflammatory activities are crucial factors in management of liver damage, so this plant is suggested to be an efficient hepatoprotective agent. In our study, the fruit extract was tested for hepatoprotective activity against liver damage induced by acetaminophen in rats. Assessment of liver function was performed by determination of its specific serum markers as well as oxidative stress. The study is also supported by histopathological studies to check any necrosis and inflammatory infiltration.

## 2. Materials and Methods

### 2.1. Animals

Seventy male adult Wistar rats of 200–250 g body weight were used in the present study. The rats had free access to food and water. They were maintained at a 12 h light and 12-h dark cycle during the experiment and were allowed to acclimatize for 1 week before starting the experiment. The animal experiments had been approved by the National Committee of Biomedical Ethics, King Abdulaziz University, Jeddah, Saudi Arabia (reference number: 163-15).

### 2.2. Drugs

Acetaminophen and silymarin were purchased from Sigma, Egypt. Silymarin was given orally once a day for one week by gavage at a dose of 100 mg/kg [13].* Adansonia digitata *extract (200 mg/kg) was given orally once daily for one week. Drugs and extract were suspended in distilled water.

### 2.3. Plant Material and Extract

The fruits were collected from Sudan and identified at Faculty of Science, King Abdulaziz University, and a voucher sample was deposited at Natural Products and Alternative Medicine Department, Faculty of Pharmacy, King Abdulaziz University, Jeddah, Saudi Arabia, under registration number AD-2014. The pulp was extracted with methanol (3 × 2000 mL) by maceration at room temperature. The combined methanol extracts were concentrated under reduced pressure and kept in refrigerator till use.

### 2.4. Acute Toxicity in Rats

To test the acute toxicity of* Adansonia digitata *extract, four groups of rats were used, six animals each.* A. digitata *extract was suspended in distilled water and administered orally once daily for one week at four different doses (200, 500, 1000, and 2000 mg/kg). Six rats served as control. During the first hour, rats were observed continuously and then every hour for 12 hours and then every day for one week, to detect any toxicity signs or mortality.

### 2.5. Acetaminophen-Induced Acute Hepatotoxicity

Acute hepatotoxicity was induced by acetaminophen (2 g/kg) orally on the fifth day, 30 min posttreatment [13, 14].

### 2.6. Experimental Design

Forty rats were divided into four groups of ten animals each (*n* = 10) and treated orally as follows:

 Group-I (normal): it was used as normal control rats and they were given distilled water orally for seven days.
 Group-II (acetaminophen): rats received distilled water orally daily for seven days; on the fifth day rats received oral dose of acetaminophen.
 Group-III (acetaminophen + silymarin): rats received silymarin (100 mg/kg) orally daily for seven days; on the fifth day rats received oral dose of acetaminophen.
 Group-IV (acetaminophen + extract): rats received extract (200 mg/kg) orally daily for seven days; on the fifth day rats received oral dose of acetaminophen.

On the seventh day, two hours after treatments [14], blood samples were obtained via retroorbital sinus plexus and then rats were sacrificed. Blood was left to clot at room temperature and the serum was obtained by centrifugation at 4000 rpm for 15 min and kept at −20°C for further biochemical analysis. Two portions of liver tissues were obtained. The first portion was immediately stored at −80°C till it was used for the different biochemical measurements, while the second portion was embedded in 10% formalin and processed for histopathological assay. Tissue sections of liver were stained with hematoxylin and eosin (H&E).

### 2.7. Measurement of Liver Function

To evaluate liver function, Alanine Amino Transaminase (ALT), Aspartate Amino Transaminase (AST), alkaline phosphatase (ALP), and total bilirubin were measured spectrophotometrically using commercial kit from BioMed Diagnostics (White City, OR, USA). Total protein was measured by a colorimetric method using a kit from Diamond Diagnostics (Cairo, Egypt) following the manufacturer's protocol.

### 2.8. Measurement of Lipid Peroxide (Measured as MDA)

MDA was determined in liver homogenates spectrophotometrically as thiobarbituric acid reactive substances (TBARS) [15].

### 2.9. Measurement of Glutathione (GSH)

GSH level was determined in the homogenates of liver following the method of Ellman [16].

### 2.10. Measurement of Superoxide Dismutase (SOD)

SOD activity was measured according to the method of Marklund [17].

### 2.11. Measurement of Catalase Activity (CATA)

The activity of catalase was measured in the homogenate of liver by spectrophotometer using CATA assay kits (Bio-Diagnostic, Egypt) as described by Aebi, 1984 [18].

### 2.12. Statistical Analysis

The obtained data were represented as mean ± standard deviation (SD). Comparisons between groups were performed using one-way analysis of variance (ANOVA) followed by Bonferroni's multiple comparison test [19]. All *P* values reported are two-tailed. *P* < 0.05 was considered statistically significant. The data were analyzed using the Statistical Package of Social Sciences (SPSS) program version 16.

## 3. Results

### 3.1. Acute Toxicity Study

After oral administration of* Adansonia digitata *extract for seven days, no mortalities were reported up to 2000 mg/kg, and hence 1/10th of the maximum dose administered (i.e., 200 mg/kg, p.o.) was selected for the present study.

### 3.2. Effect of* Adansonia digitata* Extract and Silymarin on Liver Functions Measured as ALT, AST, ALP, Bilirubin, and Total Protein

The results of the liver function tests are shown in Table 1. Administration of acetaminophen resulted in a significant (*P* < 0.05) elevation of hepatospecific serum markers ALT, AST, ALP, and total bilirubin. On the other hand, there was a significant reduction in the total protein in comparison with the normal control rats. These deleterious effects of acetaminophen were significantly (*P* < 0.05) alleviated by pretreatment with either silymarin or* Adansonia digitata *extract compared to the acetaminophen group. We did not find any significant difference between silymarin treated rats and* Adansonia digitata *extract treated rats (Table 1).

### 3.3. Effect of* Adansonia digitata* Extract and Silymarin on Acetaminophen-Induced Changes in Liver MDA, GSH, SOD, and CAT Activities

**Figure 1:**
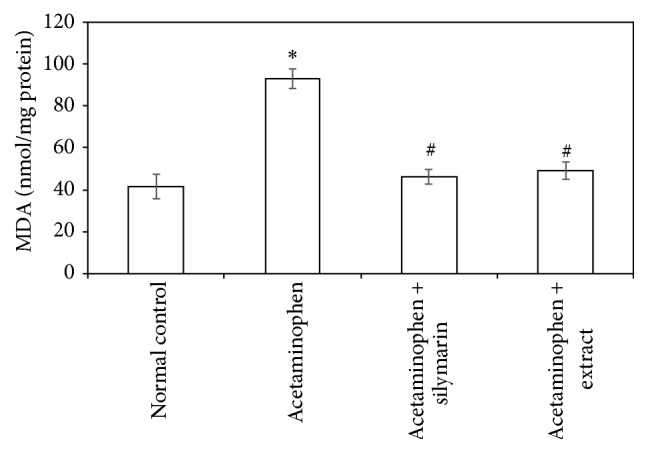
Effect of* Adansonia digitata extract *(200 mg/kg) and silymarin (100 mg/kg) on liver lipid peroxides (measured as MDA) concentration in acetaminophen-induced hepatotoxicity in rats. Each point represents the mean ± SD of ten rats.  ^*∗*^Significant difference compared with the control group (*P* < 0.05).  ^#^Significant difference compared with the acetaminophen group (*P* < 0.05).

**Figure 2 fig2:**
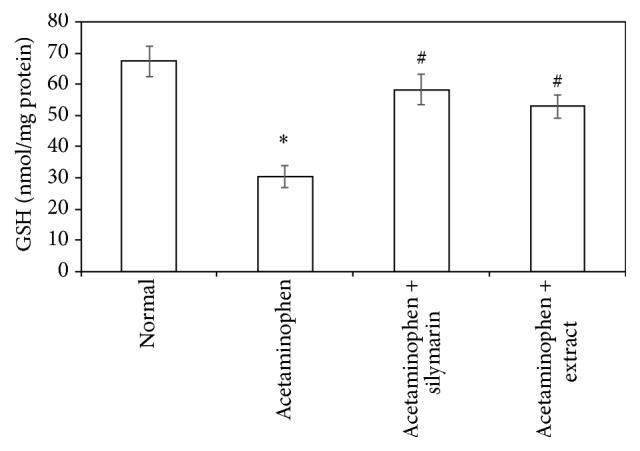
Effect of* Adansonia digitata extract *(200 mg/kg) and silymarin (100 mg/kg) on glutathione (GSH) level in acetaminophen-induced hepatotoxicity in rats. Each point represents the mean ± SD of ten rats.  ^*∗*^Significant difference compared with the control group (*P* < 0.05).  ^#^Significant difference compared with the acetaminophen group (*P* < 0.05).

**Figure 3 fig3:**
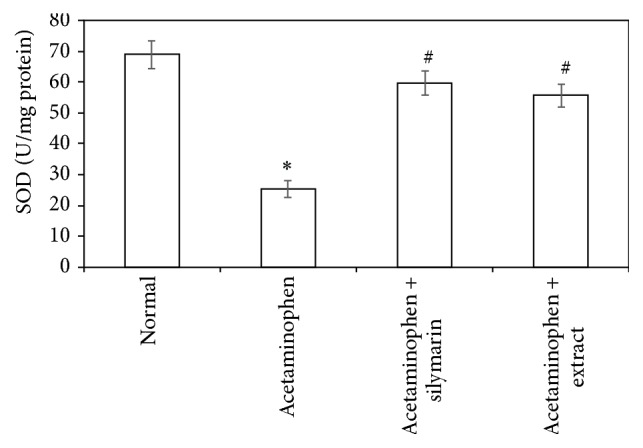
Effect of* Adansonia digitata extract *(200 mg/kg) and silymarin (100 mg/kg) on superoxide dismutase (SOD) activity in acetaminophen-induced hepatotoxicity in rats. Each point represents the mean ± SD of ten rats.  ^*∗*^Significant difference compared with the control group (*P* < 0.05).  ^#^Significant difference compared with the acetaminophen group (*P* < 0.05).

**Figure 4:**
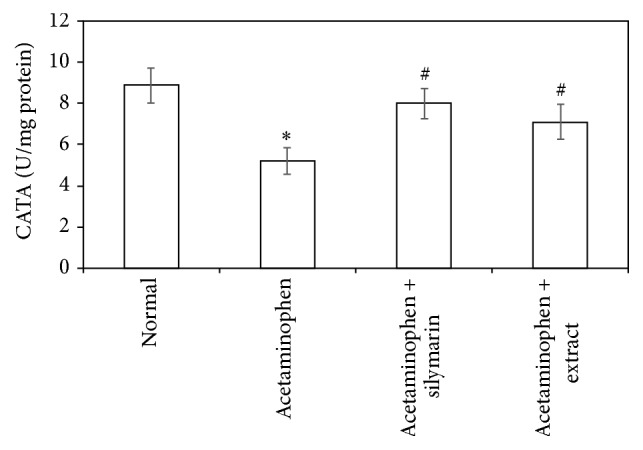
Effect of* Adansonia digitata extract *(200 mg/kg) and silymarin (100 mg/kg) on liver catalase activity (CATA) measured in acetaminophen-induced hepatotoxicity in rats. Each point represents the mean ± SD of ten rats.  ^*∗*^Significant difference compared with the control group (*P* < 0.05).  ^#^Significant difference compared with the acetaminophen group (*P* < 0.05).

Acetaminophen-induced oxidative stress in liver was in the form of significant elevation (*P* < 0.05) of MDA levels associated with significant (*P* < 0.05) reduction in SOD, GSH activities, and CATA levels compared with the normal animals (Figures 1 [Fig fig2] [Fig fig3]–4). These deleterious findings induced by acetaminophen administration were improved significantly (*P* < 0.05) upon treating the animals with either silymarin or* Adansonia digitata *extract compared to acetaminophen group. There was no significant difference between silymarin and* Adansonia digitata *extract treated groups, suggesting that* Adansonia digitata *extract has the same ameliorating effect as the standard reference drug silymarin (Figures 1–4).

### 3.4. Histopathological Microscopic Study

**Figure 5:**
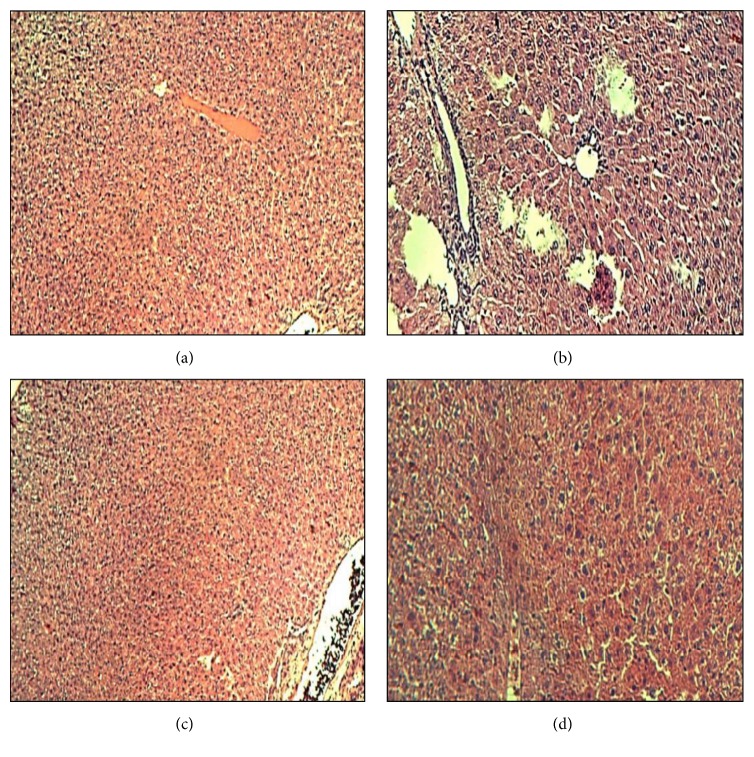
Histopathological study of liver tissue in control, acetaminophen, silymarin, and* Adansonia digitata *extract groups of rats. (a) Control group showed normal liver architecture (H&E ×40). (b) Acetaminophen group showed marked hepatic cell necrosis and moderate inflammation and lymphocytic infiltrations (H&E ×40). (c) Acetaminophen + Silymarin (100 mg/kg) showed nearly normal liver structure (H&E ×100). (d) Acetaminophen +* Adansonia digitata extract *(200 mg/kg) showed mild necrosis and inflammation (H&E ×100).

The histopathologic study of liver tissues revealed that neither necrosis nor inflammation was noticed in the normal control animals which showed normal histologic structure with integrity of hepatic cells (Figure 5(a)). On the other hand, acetaminophen treated group showed necrosis of hepatic cells, inflammation, and lymphocytic infiltrations (Figure 5(b)). Pretreatment with silymarin showed nearly normal liver structure (Figure 5(c)). Pretreatment with* Adansonia digitata *extract showed parenchyma preservation of hepatocytes with mild necrosis and inflammation (Figure 5(d)).

## 4. Discussion

Liver is a large organ responsible for metabolism, detoxification, and protein synthesis [20, 21]. Drug-induced hepatotoxicity is one of the major causes of human mortality all over the world [22]. Protection against acetaminophen-induced toxicity has been used as a test for a potential hepatoprotective agent by several investigators [23–26].

Acetaminophen is a common analgesic-antipyretic drug. It is safe in therapeutic doses. Many studies demonstrated the induction of necrosis of hepatic cells by high doses of acetaminophen in animals [2]. After high dosage of acetaminophen, it is extensively metabolized into N-acetyl-p-benzoquinoneimine (NAPQI) which depletes GSH and leads to hepatotoxicity [13, 14]. Acetaminophen is also shown to directly inhibit cellular proliferation, induce oxidative stress, resulting in lipid peroxidation, deplete ATP levels, and alter Ca++ homeostasis; all of these changes are considered potentially fatal to the cell [27, 28].

To evaluate liver injury, biochemical markers (ALT, AST, and ALP activity and serum bilirubin) levels are measured [29, 30]. In our study the hepatotoxicity due to acetaminophen was confirmed by elevated levels of biochemical parameters like ALT, AST, ALP, and total serum bilirubin with significant reduction in the total protein. This can be explained by the fact that hepatic cells contain a host of enzymes and possess a variety of metabolic activities. ALT was found in higher concentration in cytoplasm and AST particularly in mitochondria. The rise in the ALT is usually accompanied by an elevation in the levels of AST, which play a vital role in the conversion of amino acids to keto acids [31]. In hepatotoxicity the transport function of liver cells is disturbed, causing leakage of plasma membrane [32], therefore resulting in leakage of these enzymes leading to an increase in their serum level. The increased level of ALT and AST in acetaminophen-induced liver injury is an indicator of cellular leakage and loss of membrane integrity of liver cells [33]. Treatment with either silymarin or extract reversed the increased levels of ALT and AST as a result of the stabilization of plasma membrane and the repair of hepatic cell damage induced by acetaminophen [13, 27, 28].

The elevated serum level of alkaline phosphatase is due to its increased synthesis by bile canaliculi cells lining in response to the increased biliary pressure and cholestasis [27, 28]. Hyperbilirubinemia was due to excessive heme destruction and block of bile duct within the liver. Accordingly, there is a mass inhibition of the conjugation reaction and release of unconjugated bilirubin from damaged hepatocytes. Pretreatment with either silymarin or extract effectively controlled alkaline phosphatase activity and bilirubin level that point towards an enhancement in the hepatic cell secretary mechanism [2, 13, 30].

Total protein decreased level is a an indicator of liver damage due to significant fall in protein synthesis. Hypoproteinemia was observed after acetaminophen administration but the level turns towards normal upon pretreatment with either silymarin or extract.

Oxidation of lipids has been suggested for acetaminophen-induced liver injury. In agreement with previous studies [2, 13], we have found an elevation in MDA levels and depletion in antioxidant activity of SOD, CATA, and GSH. The elevated MDA in liver indicates failure of antioxidant defense mechanisms [34]. Pretreatment with silymarin or extract significantly restored these effects. The body defense mechanism prevents cell damage induced by free radicals [28]. This is established by the endogenous antioxidant enzymes, such as GSH, SOD, and CATA [35]. If there is no balance between the production of ROS and antioxidant defenses, oxidative stress results and leads to cell damage. Any compound that has antioxidant properties can prevent or alleviate this damage [28]. In our study, decreased levels of GSH, SOD, and CATA, observed in acetaminophen treated rats, are an indication of tissue damage produced by free radicals. The increase in the concentration of these antioxidant enzymes in liver tissues of silymarin- or extract-treated animals indicates antioxidant effect of silymarin and extract.

The histopathological findings confirmed the biochemical results. Rats that were treated with acetaminophen showed necrosis of hepatocytes which was manifested by disappearance of nuclei and aggregation of inflammatory cells. This may be a result of the formation of free radicals and oxidative stress induced by acetaminophen. These histopathologic findings were ameliorated significantly in the group of rats that were treated with either silymarin or* Adansonia digitata *extract.

Silymarin and* Adansonia digitata *extract showed similar hepatoprotective effect in the present study. The possible mechanism of hepatoprotection offered by silymarin is due to its high phenolic content which has been known to contribute to the antioxidant activity [2, 6, 7]. Our results on the extract are compatible with previous reports of chemical investigation of* Adansonia digitata *which reported that it accumulates flavonoids, terpenoids, steroids, amino acids, vitamins, lipids, and carbohydrates [9], while the dry fruit pulp possesses high percentage of carbohydrates, thiamine, nicotinic acid, and vitamin C. Accordingly, it is possible that the mechanism of hepatoprotective effect of extract might be due to its antioxidant effect which is attributed to its content of vitamin C and the flavonoids that are well known for potent free radical scavenging activity and enhancement of the antioxidant defense system. Moreover, the steroid content with their anti-inflammatory activity can play a significant role in the hepatoprotective effect of the extract.

## 5. Conclusions

Our study suggested a significant protective effect of* Adansonia digitata *extract against acetaminophen-induced hepatotoxicity.* Adansonia digitata *extract exerts this protection through amelioration of lipid peroxidation by its scavenging activity of free radicals and enhancement of the antioxidant defense system.

## Figures and Tables

**Table 1:** Effect of *Adansonia digitata *extract on serum liver enzymes (ALT, AST, and ALP), total bilirubin, and total protein in acetaminophen-induced liver damage in rats.

Group	Regimen	ALT (IU/L)	AST (IU/L)	ALP (IU/L)	Total Bilirubin (mg/dL)	Total Protein (mg/dL)
I	Normal	57.4 ± 0.32	80.3 ± 0.60	130.5 ± 0.25	1.27 ± 0.20	9.8 ± 0.33
II	Acetaminophen	190.3 ± 0.24^*∗*^	123.5 ± 0.74^*∗*^	220.8 ± 0.55^*∗*^	3.29 ±0.90^*∗*^	4.6 ± 0.67^*∗*^
III	Acetaminophen + silymarin	60.6 ± 0.22^#^	89.4 ± 0.29^#^	145.9 ± 0.73^#^	1.77 ± 0.48^#^	7.8 ± 0.79^#^
IV	Acetaminophen + extract	65.4 ± 0.11^#^	95.6 ± 0.82^#^	150.7 ± 0.67^#^	2.00 ± 0.60^#^	6.9 ± 0.61^#^

Results were expressed as mean ± SD and analyzed using one-way ANOVA followed by Bonferroni's post hoc test.

^*∗*^ *P* < 0.05 compared to normal control group.

^#^ *P* < 0.05 compared to acetaminophen group, *n* = 10.

## References

[1] Elberry A. A., Harraz F. M., Ghareib S. A. (2010). Antihepatotoxic effect of *Marrubium vulgare* and *Withania somnifera* extracts on carbon tetrachloride—induced hepatotoxicity in rats. *Journal of Basic and Clinical Pharmacy*.

[2] Dash D. K., Yeligar V. C., Nayak S. S. (2007). Evaluation of hepatoprotective and antioxidant activity of *Ichnocarpus frutescens* (Linn.) R.Br. on paracetamol-induced hepatotoxicity in rats. *Tropical Journal of Pharmaceutical Research*.

[3] Ahmad R., Srivastava S. P., Maurya R., Rajendran S. M., Arya K. R., Srivastava A. K. (2008). Mild antihyperglycaemic activity in *Eclipta alba*, *Berberis aristata*, *Betula utilis*, *Cedrus deodara*, *Myristica fragrans* and *Terminalia chebula*. *Indian Journal of Science and Technology*.

[4] Bent S. (2008). Herbal medicine in the United States: review of efficacy, safety, and regulation: grand rounds at University of California, San Francisco Medical Center. *Journal of General Internal Medicine*.

[5] Kintzios A., Spiridon E. (2006). Terrestrial plant derived anticancer agents and plant species used in anticancer research. *Critical Reviews in Plant Sciences*.

[6] Fraschini F., Demartini G., Esposti D. (2002). Pharmacology of silymarin. *Clinical Drug Investigation*.

[7] Kucharská J., Uličná O., Gvozdjáková A. (2004). Regeneration of coenzyme Q9 redox state and inhibition of oxidative stress by Rooibos tea (*Aspalathus linearis*) administration in carbon tetrachloride liver damage. *Physiological Research*.

[8] De Caluwé E., Halamová K., Van Damme P. (2010). *Adansonia digitata* L.—a review of traditional uses, phytochemistry and pharmacology. *Afrika Focus*.

[9] Osman M. A. (2004). Chemical and nutrient analysis of baobab (*Adansonia digitata*) fruit and seed protein solubility. *Plant Foods for Human Nutrition*.

[10] Vertuani S., Braccioli E., Buzzoni V., Manfredini S. (2002). Antioxidant capacity of *Adansonia digitata* fruit pulp and leaves. *Acta Phytotherapeutica*.

[11] Fagbohun A. A., Ikokoh P. P., Afolayan M. O., Olajide O. O., Fatokun O. A., Akanji F. T. (2012). Chemical composition and anti-oxidant capacity of the fruit pulp of *Adansonia digitata* L.. *International Journal of Applied Chemistry*.

[12] Ramadan A., Harraz F. M., El-Mougy S. A. (1994). Anti-inflammatory, analgesic and antipyretic effects of the fruit pulp of *Adansonia digitata*. *Fitoterapia*.

[13] Biswas K., Kumar A., Babaria B., Prabhu K., Setty R. (2010). Hepatoprotective effect of leaves of Peltophorum pterocarpum against paracetamol induced acute liver damage in rats. *Journal of Basic and Clinical Pharmacy*.

[14] Ramachandra Setty S., Quereshi A. A., Viswanath Swamy A. H. M. (2007). Hepatoprotective activity of *Calotropis procera* flowers against paracetamol-induced hepatic injury in rats. *Fitoterapia*.

[15] Preuss H. G., Jarrell S. T., Scheckenbach R., Lieberman S., Anderson R. A. (1998). Comparative effects of chromium, vanadium and gymnema sylvestre on sugar-induced blood pressure elevations in SHR. *Journal of the American College of Nutrition*.

[16] Ellman G. L. (1970). SH groups determination in biological fluids. *Analytical Biochemistry*.

[17] Marklund S. L. (1992). Regulation by cytokines of extracellular superoxide dismutase and other superoxide dismutase isoenzymes in fibroblasts. *The Journal of Biological Chemistry*.

[18] Aebi H. (1984). Catalase *in vitro*. *Methods in Enzymology*.

[19] Katz M. (2006). *Study Design and Statistical Analysis: A Practical Guide for Clinicians*.

[20] Ahsan R., Islam K. M., Musaddik A., Haque E. (2009). Hepatoprotective activity of methanol extract of some medicinal plants against carbon tetrachloride induced hepatotoxicity in albino rats. *Global Journal of Pharmacology*.

[21] Angelico M., Gridelli B., Strazzabosco M. (2005). Practice of adult liver transplantation in Italy: recommendations of the Italian Association for the Study of the Liver (A.I.S.F.). *Digestive and Liver Disease*.

[22] Bhawna S., Kumar S. U. (2009). Hepatoprotective activity of some indigenous plants. *International Journal of PharmTech Research*.

[23] Dwivedi Y., Rastogi R., Garg N. K., Dhawan B. N. (1991). Prevention of paracetamol-induced hepatic damage in rats by Picroliv, the standardized active fraction from *Picrorhiza kurroa*. *Phytotherapy Research*.

[24] Visen P. K. S., Shukia B., Patnaik G. K., Dhawan B. N. (1993). Andrographolide protects rat hepatocytes against paracetamol-induced damage. *Journal of Ethnopharmacology*.

[25] Singh A., Handa S. S. (1995). Hepatoprotective activity of *Apium graveolens* and *Hygrophila auriculata* against paracetamol and thioacetamide intoxication in rats. *Journal of Ethnopharmacology*.

[26] Kalantari H., Khorsandi L. S., Taherimobarekeh M. (2007). The protective effect of the *Curcuma longa* extract on acetaminophen induced hepatotoxicity in mice. *Jundishapur Journal of Natural Pharmaceutical Products*.

[27] Rabiul H., Subhasish M., Sinha S., Roy M. G., Sinha D., Gupta S. (2011). Hepatoprotective activity of *Clerodendron inerme* against paracetamol induced hepatic injury in rats for pharmaceutical product. *International Journal of Drug Development and Research*.

[28] Gini K. C., Muraleedhara K. G. (2010). Hepatoprotective effect of *Spirulina lonar* on paracetamol induced liver damage in rats. *Asian Journal of Experimental Biological Sciences*.

[29] Kozer E., Evans S., Barr J. (2003). Glutathione, glutathione-dependent enzymes and antioxidant status in erythrocytes from children treated with high-dose paracetamol. *British Journal of Clinical Pharmacology*.

[30] Girish C., Koner B. C., Jayanthi S., Rao K. R., Rajesh B., Pradhan S. C. (2009). Hepatoprotective activity of six polyherbal formulations in paracetamol induced liver toxicity in mice. *Indian Journal of Medical Research*.

[31] Sallie R., Tredger J. M., Williams R. (1991). Drugs and the liver. *Biopharmaceutics & Drug Disposition*.

[32] Rajesh M. G., Latha M. S. (2004). Preliminary evaluation of the antihepatotoxic activity of Kamilari, a polyherbal formulation. *Journal of Ethnopharmacology*.

[33] Abraham P. (2005). Oxidative stress in paracetamol-induced pathogenesis: (I). Renal damage. *Indian Journal of Biochemistry & Biophysics*.

[34] Shao H.-B., Chu L.-Y., Lu Z.-H., Kang C.-M. (2008). Primary antioxidant free radical scavenging and redox signaling pathways in higher plant cells. *International Journal of Biological Sciences*.

[35] Prakash J., Gupta S. K., Kochupillai V., Singh N., Gupta Y. K., Joshi S. (2001). Chemopreventive activity of Withania somnifera in experimentally induced fibrosarcoma tumours in swiss albino mice. *Phytotherapy Research*.

